# Cross-Reactive Antibody Responses against Nonpoliovirus Enteroviruses

**DOI:** 10.1128/mbio.03660-21

**Published:** 2022-01-18

**Authors:** Amy B. Rosenfeld, Edmund Qian Long Shen, Michaela Melendez, Nischay Mishra, W. Ian Lipkin, Vincent R. Racaniello

**Affiliations:** a Department of Microbiology and Immunology, Vagelos College of Physicians and Surgeons, Columbia University grid.239585.0grid.21729.3f , New York, New York, USA; b Center for Infection and Immunity, Department of Epidemiology, Mailman School of Public Health, Columbia University grid.239585.0grid.21729.3f , New York, New York, USA; Indiana University Bloomington

**Keywords:** antibody, antigenic variation, enterovirus, poliovirus, serotype

## Abstract

Enteroviruses are among the most common human viral pathogens. Infection with members of a subgroup of viruses within this genus, the nonpoliovirus enteroviruses (NPEVs), can result in a broad spectrum of serious illnesses, including acute flaccid myelitis (AFM), a polio-like childhood paralysis; neonatal sepsis; aseptic meningitis; myocarditis; and hand-foot-mouth disease. Despite the diverse primary sites of virus infection, including the respiratory and alimentary tracts, and an array of diseases associated with these infections, there is significant genetic and antigenic similarity among NPEVs. This conservation results in the induction of cross-reactive antibodies that are either able to bind and neutralize or bind but not neutralize multiple NPEVs. Using plaque reduction and enzyme-linked immunosorbent assay (ELISA)-based binding assays, we define the antigenic relationship among poliovirus and NPEVs, including multiple isolates of EV-D68, EV-A71, EV-D70, EV-94, EV-111, Coxsackievirus A24v, and rhinovirus. The results reveal extensive cross-reactivity among EVs that cannot be predicted from phylogenetic analysis. Determining the immunologic relationship among EVs is critical to understanding the humoral response elicited during homologous and heterologous virus infections.

## INTRODUCTION

Human enteroviruses are single-stranded (+) sense RNA viruses of the *Picornaviridae*. The enterovirus genus includes poliovirus and highly related viruses known as nonpoliovirus enteroviruses (NPEVs). More than 110 NPEVs have been identified including echoviruses, Coxsackieviruses A and B (CVA and CVB), and EVs A and D (EV-A and EV-D). These viruses are the most common human pathogens. Infection with these agents can result in a broad spectrum of serious illnesses including acute flaccid myelitis (AFM), a polio-like childhood paralysis; neonatal sepsis; aseptic meningitis; myocarditis; hand-foot-mouth disease; respiratory illness; and encephalitis. Also included within this group of viruses are the human rhinoviruses (HRVs), of which more than 160 genotypes have been identified. Development of severe respiratory complications, including pneumonia and bronchiolitis, especially in patients with chronic obstructive pulmonary disease, cystic fibrosis, and asthma, is associated with infection by both HRVs and the NPEV enterovirus D68 (EV-D68) (1–5). The global economic costs of NPEVs, including infections by human rhinoviruses, Coxsackieviruses, and echoviruses, are estimated to exceed $60 billion per year (6, 7).

Outbreaks of AFM in the United States and Europe in 2014, 2016, and 2018 were associated with increased frequency of diagnoses of EV-D68 infections, suggesting that this respiratory virus is a causative agent of childhood paralysis. Other NPEVs are potentially implicated in the development of AFM. Recent outbreaks of AFM in Brazil, Egypt, Nigeria, China, and the Democratic Republic of the Congo have been attributed to oral-fecal transmission of the related NPEVs echovirus 29, EV-B93, EV-C99, EV-D94, and EV-D111 (8–13). The surge in confirmed AFM diagnoses led the NIH to declare AFM a global epidemic in 2019 (14).

Cross-species transmission of NPEVs is suggested by the isolation of human enteroviruses from animals. EV-C99 was isolated from a chimpanzee in the Congo 1 month after an outbreak of wild poliovirus type 1 in humans in 2010. This animal had lower limb paralysis. Furthermore, echovirus 29 has been isolated from the stool of nonhuman primates in Cameroon (8, 12), and humans were implicated in an outbreak of rhinovirus C that took place in a colony of chimpanzees (15).

The presence of antibodies against a pathogen is frequently taken as evidence of prior infection or exposure to that agent. However, this conclusion does not reflect the potential of antibody cross-reactivity, whereby antibodies generated against one pathogen can bind to a heterologous pathogen. For instance, the observation that most adults are seropositive for antibodies against enteroviruses including EV-D68 has been used as evidence of prior infection with this virus (16–19). However, few EV-D68 infections were reported between 1962 and the 2014 outbreak, and it has been suggested that antibodies to other enteroviruses are able to bind the EV-D68 particle (20). Accordingly, the high apparent prevalence of EV-D68 infection based on serology may reflect reactivity. There is precedent for cross-reactive enterovirus antibodies. One study determined that antibodies generated against a peptide derived from the N terminus of the VP-1 capsid protein of poliovirus type 1/Mahoney can bind Coxsackievirus B3 and EV-D70, while antibodies elicited against the VP-1 capsid protein of EV-A71 interact with Coxsackievirus A16 (21–27). These cross-reactive antibodies are thought to bind but not neutralize the heterologous pathogen.

Another misleading common concept is that monoclonal antibodies to individual serotypes of a virus cannot bind and/or neutralize other serotypes. This postulate is used to define serotype and explain why infection with one serotype of poliovirus confers minimal protection against infection with viruses of the other two serotypes. While poliovirus serotypes are defined by neutralization assays, the lack of cross-neutralization is not absolute: individual serotypes may be incompletely neutralized by antibodies raised against the other two serotypes (28), suggesting that antibodies able to neutralize multiple strains of poliovirus can be identified. Results from studies done in the 1920s and 1930s by Stewart and Rhoads, Aycock, and Burnet and Macnamara in which monkeys were infected with either the 1909 “MA” isolate of poliovirus from a fatal case of poliomyelitis, the Vermont “Aycok” isolate from 1920, or the Australian “Victoria” virus identified in 1928 and challenged with infection by a heterologous isolate provide evidence for some cross-protection among serotypes (29–31). Additionally, monospecific polyclonal sera from seven monkeys, each immunized with an individual untyped viral isolate from patients during the 1949 poliomyelitis outbreak in Kansas City, were found to partially neutralize more than one prototype poliovirus (32). Sera from four animals neutralized type 1 and 2 poliovirus, one serum neutralized type 2 and 3 poliovirus, and two sera neutralized type 1 and 3 poliovirus. A more recent example of an antibody that can bind virus particles of multiple poliovirus serotypes is the human monoclonal antibody A12 (33). This monoclonal antibody binds all three serotypes of poliovirus, albeit with different affinities and at slightly different sites on the virus particle. This antibody also blocks infection of cells in culture by poliovirus serotypes 1 and 2 but not serotype 3. One possible explanation for why these observations apparently contradict the concept of serotype is immune dominance of an epitope. The peptide recognized by A12 monoclonal antibody may not be the immune dominant epitope recognized by antibodies that block infection by poliovirus serotype 3. Structural analysis of the A12-type 1 poliovirus interaction revealed that the antibody interacts with amino acids located on opposing walls of the canyon. These amino acids are found within two known neutralizing sites, sites 1 and 2, but differ slightly among the serotypes (33). Serotype specificity of the physical interaction between monoclonal antibody (MAb) A12 and poliovirus is similar to that between the virus receptor and the virion. CD155, the human poliovirus receptor, binds within the canyon of all three serotypes of poliovirus; however, alterations within the virus binding site of CD155 reduce its affinity for poliovirus in a serotype-specific manner (34). This observation suggests that subtle differences in structure of the virus can lead to significant changes in atomic interactions between proteins.

Additional evidence for the ability of cross-serotype anti-enterovirus antibodies to block infection stems from studies done in the 1960s when volunteers were intranasally infected with HRV strains NIH 1734 and 353 (types 8 and 23, respectively). When volunteers were challenged with the heterologous virus 2 weeks after primary inoculation, no disease was observed (35). Furthermore, murine antibodies against the N terminus of the VP-4 capsid protein of HRV-A14 can bind HRV-A16 and HRV-A29 (36). These interactions prevent infection of cells in culture by the two heterologous serotypes of HRV. Moreover, polyclonal sera from mice and rabbits immunized with recombinant VP-1 capsid protein from either HRV-A89 or HRV-B14 were able to block infection of cells in culture by multiple HRVs, including HRV-A1A, HRV-A3, and HRV-A72 (37).

Today, genomic analysis instead of serology is used to define different variants and genotypes of a virus. Results of studies examining both polyclonal human sera and a recombinant monoclonal antibody against the VP-4 capsid protein of EV-A71 suggest that antibodies against one genotype of EV-A71 can block infection of cells in culture by other genotypes and serotypes of the virus, as well as Coxsackievirus A16 and A6 (38, 39). Antibody cross-reactivity among enteroviruses has also been observed using peptide arrays derived from the capsid proteins of multiple enteroviruses, including EV-D68, poliovirus type 1/Mahoney, Coxsackievirus A4 and B1, and human rhinoviruses (26, 40, 41). Whether or not cross-reactive antibodies can neutralize viruses of different enterovirus species (e.g., EV-C and EV-D) has not been demonstrated.

To provide additional insight into the cross-reactive antigenic structure of enteroviruses, we explored the ability of antibodies against multiple EV species A to D and HRVs to bind and cross-neutralize infectivity. Plaque reduction and enzyme-linked immunosorbent assay (ELISA)-based binding assays were used to define the antigenic relationship among poliovirus and NPEVs, including multiple isolates of EV-D68, EV-A71, EV-D70, EV-94, EV-111, Coxsackievirus A24v, and rhinovirus. The results reveal extensive cross-reactivity among enteroviruses that cannot be predicted from phylogenetic analysis and that antigenic groups exist among this genus of viruses. Determining the immunologic relationship among enteroviruses is critical to understanding the humoral responses elicited during homologous and heterologous virus infections.

## RESULTS

### Cross-neutralization of human anti-NPEV antibodies.

ELISA binding assays have previously been to test for the presence of cross-reactive anti-enterovirus antibodies in human polyclonal sera (16–19, 26, 40–42). Only EV-D68 was used in antibody neutralization assays in some of these studies. The results of these studies led to the hypothesis that the human population may be continuously infected by EV-D68 despite few cases of EV-D68 reported in the United States and Europe prior to the 2014 outbreak. An alternative explanation for these observations is that antibodies elicited during enterovirus infection may neutralize heterologous enteroviruses. While modeling studies suggest that the global incidence most of enterovirus infections can be explained by acquired serotype-specific immunity, homotypic immunity does not explain the increased number of CVA-6 and EV-B18 (echovirus 18) infections (43). Instead, it has been suggested that evolution in antigenic and/or other regions or a cross-reactive immune response may account for the rise in global incidence of CVA-6 and EV-B18 (43). To confirm the presence of anti-enterovirus antibodies, specifically against EV-D68 and EV-A71, in human polyclonal sera pooled from nine adults not known to have prior EV-D68 or EV-A71 infections, sera were incubated with 10^5^ PFU of multiple isolates of EV-D68, an isolate of EV-A71 obtained in 2018 and associated with AFM, and poliovirus type 1/Mahoney. As anticipated, the highest neutralization activity was observed against poliovirus, presumably because most Americans have been vaccinated against this virus. Neutralization of EV-A71 and two isolates of EV-D68 was also observed albeit at different efficiencies (Table 1). These data suggest that neutralizing antibodies against multiple enteroviruses are present in healthy human sera.

**TABLE 1:** Neutralization of poliovirus type 1/Mahoney, EV-D68, and EV-A71 by pooled polyclonal human sera^*a*^

Virus	Titer^*b*^
Poliovirus P1/Mahoney	2,048
EV-D68, NY	128
EV-D68, 23216	64
EV-A71	256

a Two-fold serial dilutions of human sera from nine healthy adults were incubated with 10^5^ PFU of each virus for 1 h at room temperature, and neutralization of infectivity was assessed by plaque reduction assay. The results are representative of three independent experiments using sera pooled from nine healthy adults.

*^b^*Highest dilution at which neutralization is observed.

### Generation of murine anti-NPEV polyclonal sera.

The data described above are unable to distinguish neutralizing antibodies induced by undiagnosed prior enterovirus infections or elicited in response to the poliovirus vaccine or that antibodies induced by a prior enterovirus infection can bind and neutralize a wide range of enteroviruses. To answer this question, polyclonal sera against a single enterovirus is required. To produce individual monospecific murine anti-enterovirus polyclonal sera, 3-week-old wild-type C57/Black6 mice were intraperitoneally immunized with 10^5^ PFU of single members of a diverse group of enteroviruses, including three isolates of EV-D68, poliovirus, EV-A71, EV-D94, and CVA-24v. Mice were bled 14 days later, and monospecific polyclonal sera were collected by centrifugation and assayed for the presence of neutralizing antibodies against the immunizing virus by plaque reduction assay. No neutralization for poliovirus was observed (Table 2). The absence of anti-enterovirus antibodies in the other monospecific anti-enterovirus sera was also observed by ELISA binding assays (data not shown). These data suggest that more than 14 days is necessary to detect anti-enterovirus antibodies in murine polyclonal sera. Subsequently, the mice were boosted every 14 days with 10^5^ PFU of the appropriate enterovirus mixed with Freund’s complete adjuvant and were bled 10 days later. This process was repeated for 2 months. Similar protocols have been previously used for the generation of anti-enterovirus polyclonal sera (44). Homotypic anti-enterovirus neutralizing antibodies were detected after the second and all subsequent bleeds (Table 2).

**TABLE 2:** Neutralization of poliovirus type 1/Mahoney by polyclonal sera from consecutive bleeds from mice immunized with poliovirus type 1/Mahoney^*a*^

Serum dilution	First bleed	After first adjuvanted boost	After second adjuvanted boost
No serum	3,500	3,350	3,000
1:2	1,200	250	0
1:4	2,650	1,030	0
1:8	3,900	1,100	0
1:16	2,200	2,600	0
1:32	4,500	160	0
1:64	3,000	240	0
1:128	3,200	550	0
1:256	3,450	1,500	0
1:512	5,100	1,800	190
1:1,024	4,250	1,500	2,100
1:2,048	4,350	1,150	1,870
1:4,096	4,450	2,650	3,300
1:8,192	3,600	1,350	1,600
1:16,384	4,450	2,750	3,700
1:32,768	4,250	2,050	1,150

a Polyclonal sera were collected from five wild-type mice 14 days after the initial immunization (first bleed) and 10 and 24 days after boosting with virus adjuvanted in Freund’s complete. Two-fold serial dilutions of polyclonal sera were incubated with 10^5^ PFU of poliovirus type 1/Mahoney for 1 h at room temperature, and neutralization of infectivity was assessed by plaque reduction assay. The results are representative of three independent experiments. The values are shown in PFU/mL.

### Cross-reactivity of murine anti-enterovirus antibodies.

**FIG 1:**
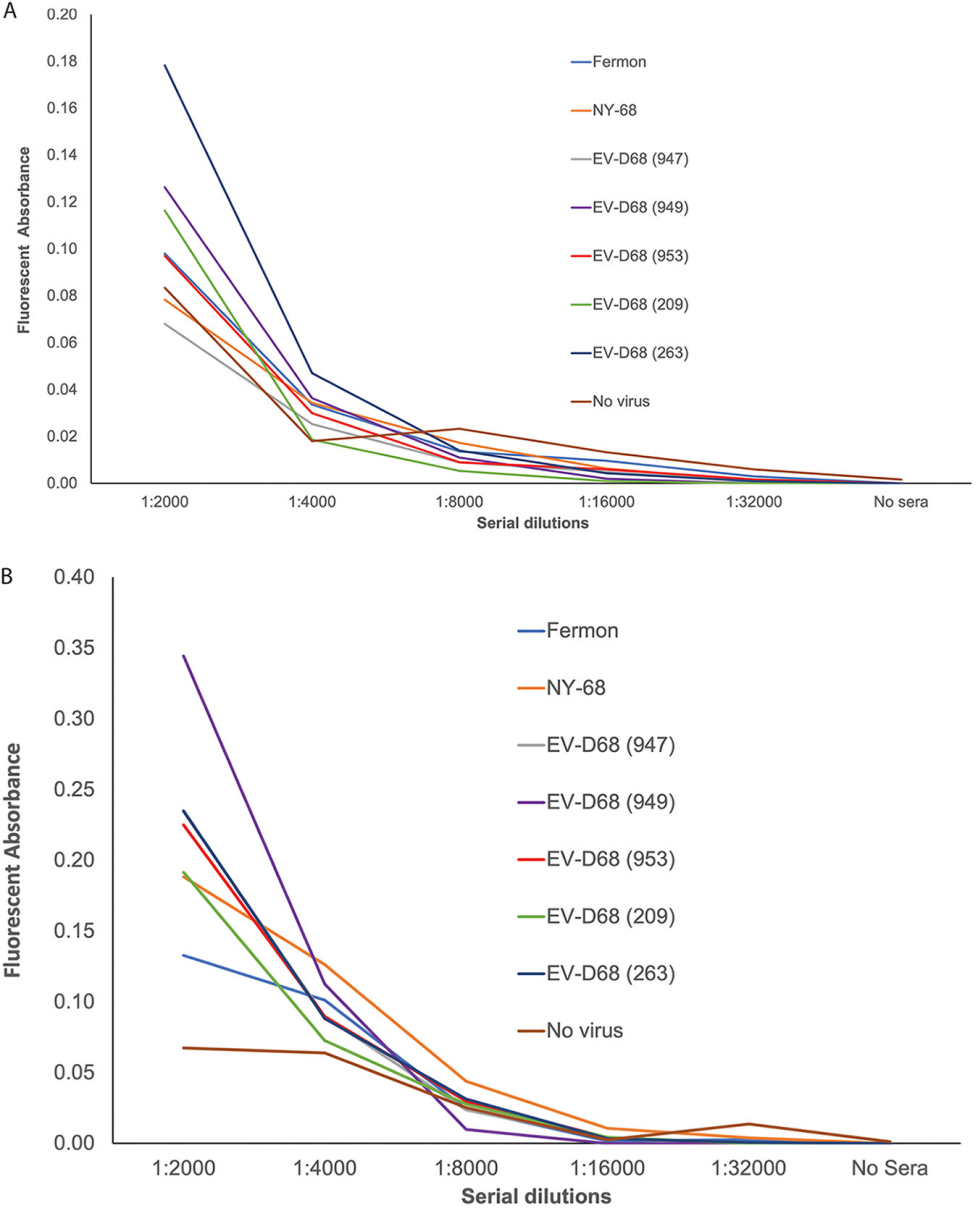
Binding of multiple isolates of EV-D68 by polyclonal sera from the sixth bleed of mice immunized with poliovirus type 1/Mahoney. Two-fold serial dilutions of polyclonal sera from mice inoculated with the poliovirus were incubated with 10^5^ PFU of multiple isolates of EV-D68 that were either directly bound to the plastic surface (A) or captured by a recombinant human monoclonal antibody specific for EV-D68 (B) (45) overnight at 4°C, and binding was assessed by enzyme-linked immunosorbent assay (ELISA) and reported as the difference between excitation (470 nm) and emission (520 nm). The results are representative of three independent experiments.

To determine whether anti-enterovirus antibodies are specific or promiscuous with respect to binding, polyclonal sera from mice that were immunized with poliovirus type 1/Mahoney were analyzed by ELISA. Sera were incubated with 10^5^ PFU of multiple isolates of EV-D68 including the Fermon and two isolates from the 2018 outbreak associated with AFM (Fig. 1). Binding was observed with the Fermon, two isolates from the 2014 outbreak, and two isolates from the 2018 outbreak associated with AFM.

One explanation for these observations is that binding of enterovirus particles to plastic wells as part of the ELISA leads to conformational changes in virion structure that expose conserved internal epitopes. To examine this possibility, an anti-EV-D68 recombinant monoclonal antibody (45) was attached to the plastic prior to the addition of EV-D68 virus particles and incubation in the presence of the anti-poliovirus sera. This procedure would eliminate any conformation changes caused by attachment of virus to the plastic. Under these conditions, interaction between EV-D68 and anti-poliovirus antibodies was still observed (Fig. 1).

**FIG 2:**
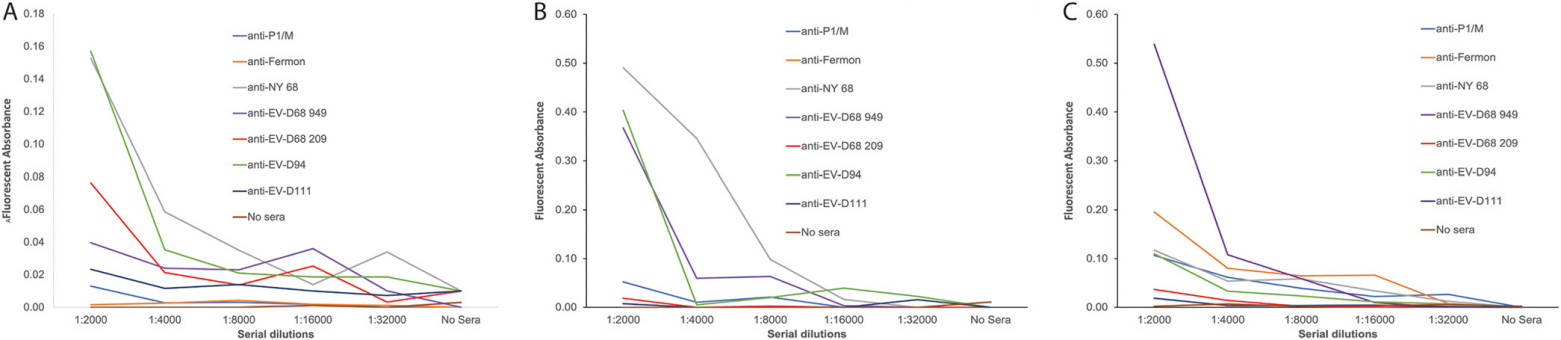
Binding of poliovirus type 1/Mahoney by polyclonal sera from mice immunized with EV-D68, EV-D94, or EV-D111. Two-fold serial dilutions of monospecific polyclonal sera from mice inoculated with the EV-D68, EV-D94, or EV-D111 were incubated with 10^5^ PFU of poliovirus type 1/Mahoney that was either directly bound to the plastic surface (A) or captured by anti-poliovirus type 1/Mahoney polyclonal rabbit sera (B) or human monoclonal antibody A12 that binds all polioviruses (C) (45) overnight at 4°C, and binding was assessed by ELISA and reported as the difference between excitation (470 nm) and emission (520 nm). The results are representative of three independent experiments.

Conversely, monospecific polyclonal sera from mice individually immunized with EV-D68 isolates from 1962 to 2018, EV-D94 or EV-D11, were incubated in the presence of 10^5^ PFU of poliovirus type 1/Mahoney, and binding was assayed by ELISA. As above, virus was both directly bound to the plastic wells or captured using polyclonal rabbit sera or recombinant anti-poliovirus monoclonal antibodies A12. Interaction between poliovirus type 1/Mahoney and monospecific polyclonal sera from mice individually immunized with EV-D68 isolates from 2009, 2014, and 2018 or with EV-D94 was observed (Fig. 2). In all cases, binding between poliovirus type 1 Mahoney and monospecific anti-poliovirus type 1 Mahoney antisera was not higher than observed when using antisera generated against other enteroviruses. In contrast, neutralization tier of anti-poliovirus type 1 Mahoney antisera was highest against the homologous virus (Table 3). The explanation for these observations is unknown, but the finding is reproducible. No interaction with monospecific polyclonal sera from immunized with an EV-D68 from 1962 or EV-D111 was detected. No binding was observed with CVB-3, CVB-5, CVA-24v, CVA-16, or HRV-A2 (Table 4). In this experiment, the pattern of interaction of antisera against various enteroviruses with poliovirus type 1/Mahoney appears to be influenced by conformational changes induced by the capture antibody used. However, the observation that antisera raised against different enteroviruses can bind poliovirus type 1/Mahoney remains unchanged.

**TABLE 3:** Neutralization of enteroviruses by monospecific polyclonal sera from bleeds from mice immunized with poliovirus type 1/Mahoney, enterovirus D68 or A71^*a*^

Virus	Anti-poliovirus type 1 sera, titer^*b*^	Anti-EV-D68 sera, titer^*c*^	Anti-EV-A71 sera, titer^*d*^
Poliovirus P1/Mahoney	>32,768	4,096	32
EV-D68, NY	1,024	16,384	<2
EV-D68, Rhyne	<2	ND	ND
EV-D68, 23216	128	ND	ND
EV-D68, IUHO4	16	ND	ND
EV-A71	32	ND	256
EV-D68, Fermon	<2	ND	ND
EV-B1	<2	ND	ND
EV-D70	<2	<2	ND
EV-D94	2	<2	<2
CAV-24v	<2	ND	ND
CVB3	<2	ND	<2
HRV-A1A	16,384	ND	<2

a Polyclonal sera were collected from 5 wild-type mice immunizing mice with either poliovirus type 1/Mahoney, EV-D68 or EV-A71 adjuvanted in Freund’s complete. Two-fold serial dilutions of polyclonal sera were incubated with 10^5^ PFU of multiple NPEVs 1h at room temperature and neutralization of infectivity was assessed by plaque reduction assay. Results are representative of three independent experiments. The values shown are the highest dilutions at which neutralization is observed.

b Results using murine anti-poliovirus type 1/Mahoney sera, bleed 5.

c Results using murine anti EV-D68, 23209 sera, bleed 4.

d Results using murine anti EV-A71, 23092 sera, bleed 2.

**TABLE 4:** Binding of antibodies within polyclonal sera from mice immunized with either poliovirus type 1/Mahoney or NY-68 isolate of EV-D68^*a*^

Virus	Binding titer of murine anti-poliovirus polyclonal sera^*b*^	Binding titer of murine anti-NY-68 polyclonal sera
Poliovirus P1/Mahoney	256	1,024
EV-D68, NY	512	256
EV-D68, 209	256	64
EV-D68, Fermon	<2^*c*^	<2
EV-D68, Rhyne	<2	<2
EV-A71, 23092	64	32
CVB 3	<2	<2
CVB 5	<2	<2
CAV 24v	ND	<2
CAV A16	ND	<2
HRV-A2	<2	<2
HRV-A1A	1,024	<2

a Polyclonal sera were collected from five wild-type mice 10 days after the fourth boost with poliovirus type 1/Mahoney or the NY 68 isolate of EV-D68 adjuvanted in Freund’s complete. Antibody binding was determined by enzyme-linked immunosorbent assay (ELISA); two-fold serial dilutions of polyclonal sera from either poliovirus type 1/Mahoney or EV-D68 were incubated with 10^5^ PFU of multiple NPEVs for 1 h at room temperature. Binding was assessed by ELISA and is reported as the difference between excitation (470 nm) and emission (520 nm). The results are representative of three independent experiments. The results using sera pooled from five mice. Readings above the fluorescence absorbance of no virus negative control (0.05) were considered positive. ND, not determined.

b Highest dilution at which binding is observed.

c No binding observed.

**FIG 3:**
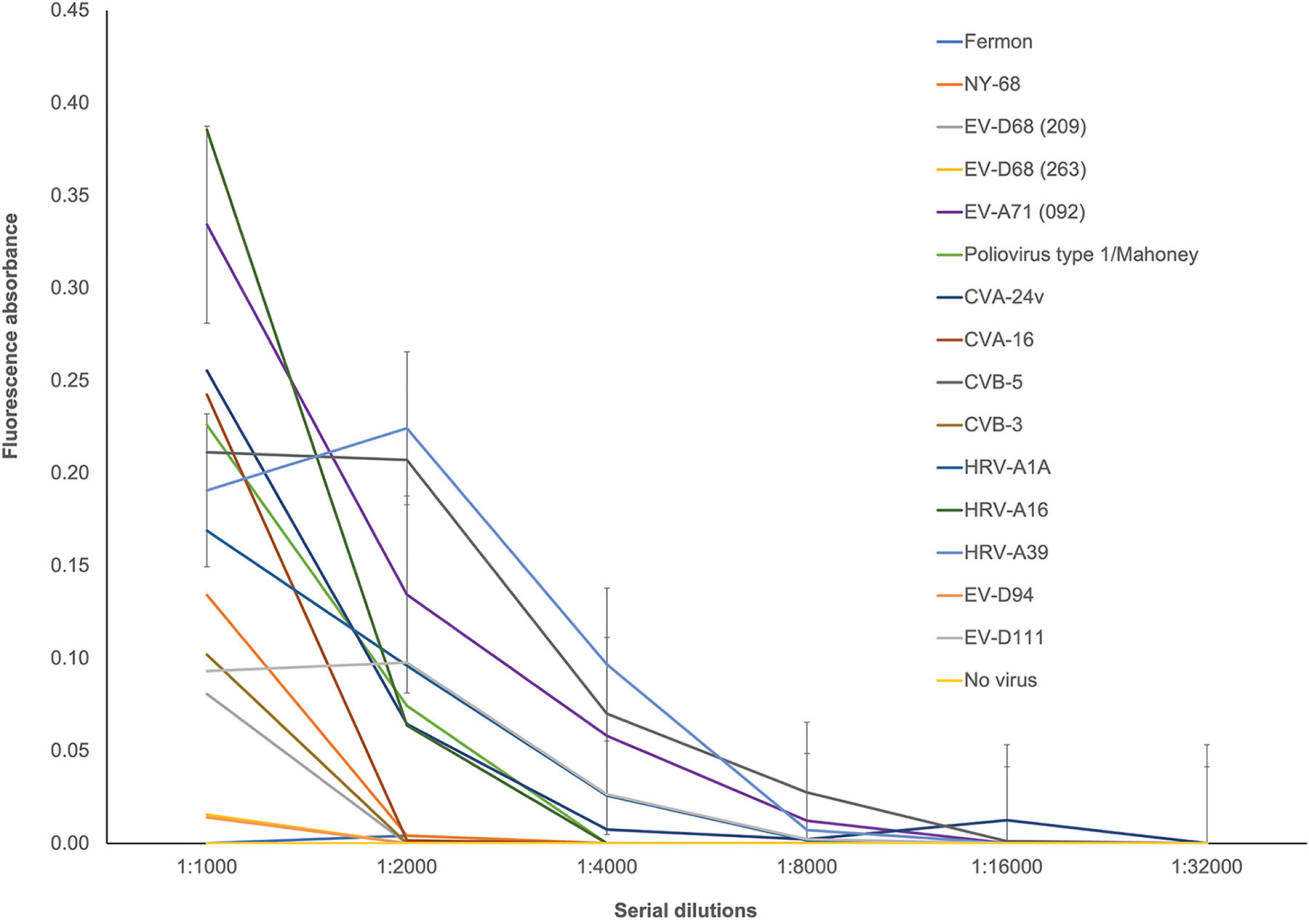
Binding of representative EVs by guinea pig polyclonal sera from animals immunized with poliovirus type 1/Mahoney. Two-fold serial dilutions of polyclonal sera from guinea pigs immunized with poliovirus type 1/Mahoney were incubated with 10^5^ PFU of NPEVs representing all species A to D and rhinoviruses. After incubation for 1 h at room temperature, binding was assessed by ELISA and reported as the difference between excitation (470 nm) and emission (520 nm). The results are representative of three independent experiments.

To test the possibility that the presence of cross-reactive anti-EV antibodies is only a characteristic of murine sera, anti-poliovirus type 1/Mahoney guinea pig sera were incubated with a set of diverse EVs including EV-D68, EV-A71, CVA-24v, CVA-16, CVB-5, CVB-3, EV-D94, EV-D111, HRV-A1A, and HRV-A39. Binding was detected with guinea pig anti-poliovirus type 1/Mahoney antibodies and isolates of EV-D68 from 2009 and two isolates associated with AFM from the 2018 outbreak, as well as with CVA-24v, CVB-3, HRV-A39, HRV-A1A, HRV-A16, EV-D111, and CVA-16 (Fig. 3). The interaction between anti-poliovirus antibodies and a heterologous EV is not due to nonspecific binding as no interaction was observed between anti-poliovirus type 1/Mahoney antibodies and CVB-5, EV-D94, or the Fermon isolates of EV-D68 (Fig. 3; Table 4).

### Anti-enterovirus antibodies inhibit infections by heterologous enteroviruses.

To determine whether the cross-binding anti-enterovirus antibodies can block virus infection of cells in culture, plaque reduction assays were performed using monospecific murine polyclonal anti-EV-D68, anti-poliovirus type 1/Mahoney sera, and anti-EV-A71 sera. Serial dilutions of anti-enterovirus murine polyclonal sera were incubated with a set of representative EVs including poliovirus type 1/Mahoney, three isolates of EV-D68, the EV-A71 isolate used previously, CVA-24v, HRV1A, and EV-D94. Sera from mice immunized with the EV-D68 isolate from the 2018 outbreak associated with AFM not only protected cells from infection by the immunizing virus but also from infection by poliovirus type 1/Mahoney but not EV-A71, EV-D94, or the DAF adapted variant of EV-D70 (Table 3). Sera from mice immunized with poliovirus type 1/Mahoney protected cells against infection by the NY-68 isolate of EV-D68, a clinical isolate from the 2014 outbreak associated only with respiratory disease, an AFM-associated isolate of EV-A71, and HRV1A. These anti-poliovirus sera weakly neutralized EV-D94 and had no activity against the Fermon and Rhyne isolates of EV-D68 or CVB3 (Table 3).

**FIG 4:**
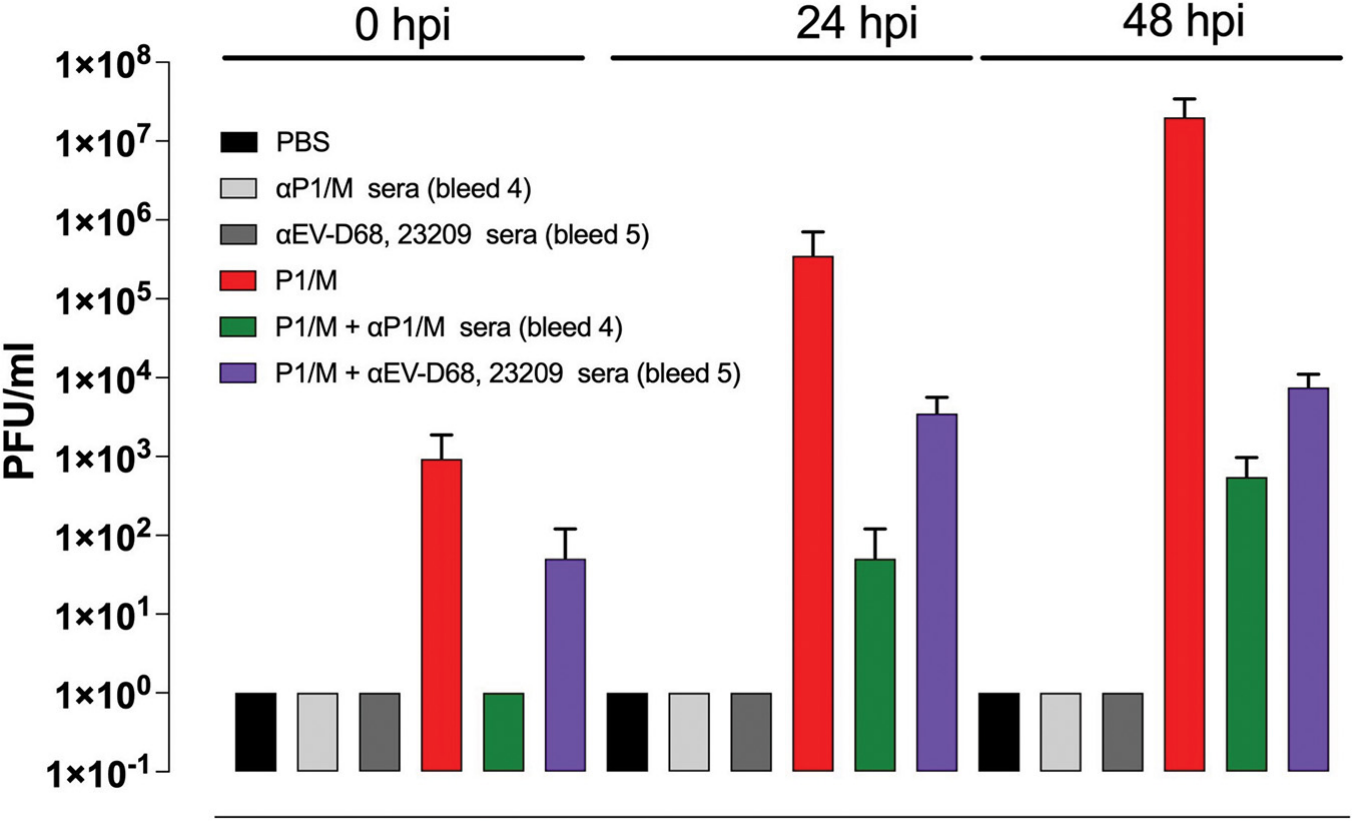
Inhibition of poliovirus type 1/Mahoney infection of murine fibroblasts producing the human poliovirus receptor by monospecific polyclonal anti-EV-D68 murine sera. Polyclonal sera were pooled from five wild-type mice immunized with an EV-D68 isolate from the 2018 outbreak that associated with AFM adjuvanted in Freund’s complete. Anti-EV-D68 or anti-poliovirus type 1/Mahoney monospecific polyclonal murine sera were diluted 1:1,024, incubated with 10^3^ PFU of poliovirus type 1/Mahoney 1 h at room temperature, and overlaid onto a monolayer of murine fibroblasts producing the human poliovirus receptor. Cells and supernatant were collected at 0, 24, and 48 h postinfection, and neutralization of infectivity was assessed by plaque reduction assay. The results are representative of three independent experiments. PBS, phosphate-buffered saline.

To confirm the observation that monospecific murine polyclonal anti-EV-D68 antisera could protect cells in culture from poliovirus type 1/Mahoney infection, murine fibroblasts that produce the human poliovirus receptor (hPVR) were infected with poliovirus type 1/Mahoney that had either been incubated in the presence of sera from wild-type mice or 2-fold serial dilutions of sera from mice immunized with an isolate of EV-D68 from the 2018 outbreak that associated with AFM or from mice immunized with poliovirus type 1/Mahoney. The cells and supernatants were collected at various times postinfection, and virus reproduction was assessed by plaque assay. Sera from mice immunized with either isolate of EV-D68 protected murine fibroblasts producing the hPVR from poliovirus infection, albeit with lower efficiency than sera from poliovirus immunized mice (Fig. 4). These observations support our hypothesis that anti-enterovirus cross-reactive antibody response can neutralize enteroviruses of different species. In addition, these data suggest that a mechanism of neutralization by cross-reactive antibodies may block the receptor-virion interaction.

## DISCUSSION

The binding sites for antibodies that neutralize virus infectivity have been identified in the three-dimensional structures of poliovirus and several EVs. The EV virus particle is a small icosahedral nonenveloped capsid comprising 60 copies of the four virus specific proteins VP1, VP2, VP3, and VP4 (reviewed by Rosenfeld and Racaniello [46]). The outer surface of the EV particle is composed of capsid proteins VP1, VP2, and VP3, while the VP4 protein lies on the internal surface of the capsid and interacts with the RNA genome. The protomer, the structural unit, is built with one copy of each capsid protein. Five protomers comprise one pentamer, and assembly of 60 pentamers gives rise to the signature 5-, 3-, and 2-fold axes of symmetry of the icosahedral particle. Five molecules of VP1 line the particle’s 5-fold axes, while VP2 and VP3 alternate at the 3-fold axes.

The structures of the surface proteins VP1 to VP3 are similar, i.e., an eight-stranded antiparallel β-barrel core. The N termini of the proteins are found within the interior of the particle, and tiling of the barrel cores results in radial protrusions at each axis. Those protrusions at the 5-fold axis are separated by a deep depression, known as the canyon (47–49), the site of receptor binding for some picornaviruses (reviewed by Rosenfeld and Racaniello [46]). The corrugated surface of the virion results from the solvent exposed loops of varied length that link the antiparallel β-strands of each barrel. These loops (BC, HI, DE, FG, GH, and CD) confer the main structural differences among EVs. For example, the distinct structural arrangement of the VP1 BC, DE, EF, and HI loops of EV-A71, CVA-16, and CVA-6 controls differences in receptor utilization and entry of these viruses but also determines the numerous unique surfaces of the CVA-6 particle (50). Many of the solvent-exposed loops are antigenic sites, i.e., targets of an antibody response.

Despite the array of diseases that result from enterovirus infections, and the diverse mechanisms of cell attachment, entry, and uncoating of enteroviruses, only four discrete neutralization antigenic sites on the viral capsid have been identified with the exception of rhinovirus C, for which two antigenic sites have been identified (reviewed by Hogle and Filman [51]). Binding of antibody to the four known neutralization antigenic sites is hypothesized to block receptor attachment. Site 1 is near the 5-fold axis and includes the top of a large loop that links the B and C strands of VP1 and a single amino acid from strand D and the DE loop. This site is located on the north rim of the canyon. Site 2, near the 2-fold axes, is the southern wall of the canyon. It is composed of two separate polypeptides derived from VP2 and the EF and GH loops of VP1. Site 3 is in and around the 3-fold axis. It is defined by residues within strand B and the BC loop of VP3, as well as a single amino acid in the BC loop of VP2. Site 4 is within the canyon. Although we do not know which sites the antibodies in the murine polyclonal sera reported here are directed against, it is likely that they will bind a minimum of one of these four antigenic sites. Conversely, we also anticipate that some antibodies in these sera will bind novel sites on the viral capsid and might mediate protection against disease via mechanisms that are dependent upon the presence of other components of the immune system, i.e., Fc receptors on certain cell types.

The genetic similarity between enteroviruses and the conserved locations of antigenic sites on the virus particle may partially explain the cross-reactive humoral response found in human sera (40, 41, 52). However, few broadly cross-neutralizing anti-EV antibodies have been identified. To address this dichotomy, we generated virus-specific murine polyclonal sera for poliovirus, EV-D68, and EV-A71 and tested for cross-binding and cross-neutralization with heterologous enteroviruses among all species, including A to D and multiple rhinoviruses. The finding that guinea pig antiserum against poliovirus neutralizes EV-D68 and EV-A71 reinforces our observations with murine antisera.

**FIG 5:**
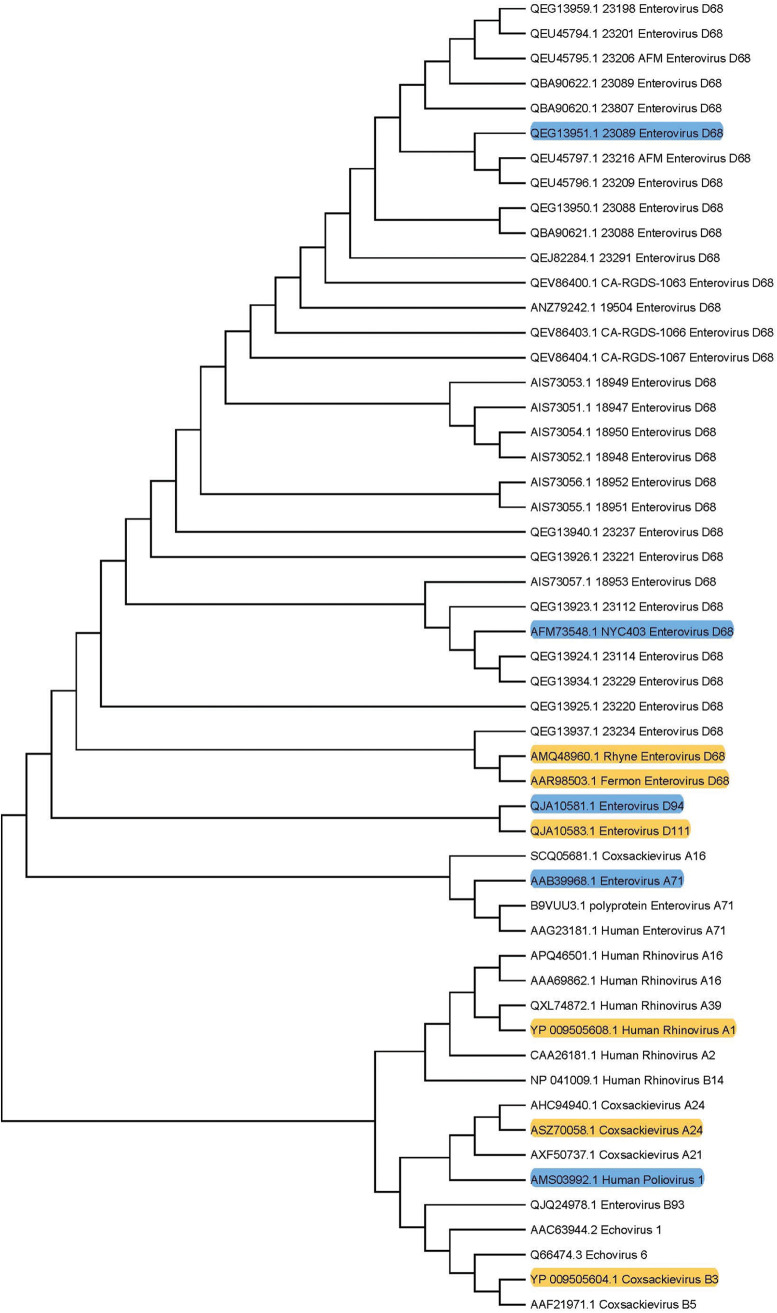
Phylogenetic analysis of the capsid protein precursor (VP1 to VP4) of enteroviruses. Total collection includes 35 isolates of EV-D68, 3 human rhinoviruses (HRVs), and 6 enteroviruses of other species from EV-A, EV-B, EV-C, and EV-D. Enterovirus D-68 samples are further classified by clades A, B, and C. Phylogenetic analysis was conducted using a maximum likelihood model after applying ClustalW sequence alignment to all genome polypeptides with the MegaX program. Viruses highlighted in blue were found to be viruses that bound to anti-poliovirus type 1/Mahoney polyclonal sera. No binding was observed with the viruses highlighted in yellow.

The identity of the antigenic sites on the viral capsid recognized by cross-reactive antibodies is currently unknown, but experiments are in progress to determine their locations and the mechanisms by which antibodies that bind these sites block infection. Phylogenetic analysis of the EV capsid proteins does not reveal the nature of such antigenic sites, including their amino acid sequence, composition, and structure (Fig. 5). The inability to predict antigenic cross-reactivity using phylogenetic analysis has been suggested to explain the properties of antibodies that cross-react with the spike protein of severe acute respiratory syndrome coronavirus (SARS-CoV) and SARS-CoV-2. These antibodies do not recognize the S2 domain of spike wherein the two viruses have 90% amino acid identity but are instead directed against the receptor-binding domain that has 73% amino acid identity (53).

Our observations have implications for understanding the prevalence of circulating enteroviruses and disease. The results of several serological studies have been interpreted to suggest that over 90% of adults have been infected with EV-D68 (9, 16–19). A subset of these studies was done in pregnant women in Finland and showed that seropositivity for EV-D68 and EV-D94 went hand in hand. Conversely, comparison of commercially available IVIG from the United States, Europe, and Asia revealed that anti-EV antibodies within these sera may reflect the geographic distribution of circulating viruses (54, 55). Consequently, we must consider the possibility that estimates of the number of adults who have been infected with EV-D68 are inflated due to the presence of the EV cross-reactive antibodies reported here and the observation that anti-enterovirus antibodies can bind the EV-D68 particle (20). Despite the presence of anti-enterovirus cross-reactive antibodies in sera from healthy individuals, it is thought that humans get one enterovirus infection a year. Our data suggest that enteroviruses may form antigenic groups, and no single antibody will be able protect against infection by all enteroviruses. Furthermore, the durability of the humoral response against enterovirus infection varies. It is thought that a protective antibody response elicited by the oral poliovirus vaccine lasts greater than 18 years, while the humoral response against human rhinoviruses A and B is far less durable (35, 56–58).

In summary, this initial report describes a pan-EV humoral response within virus-specific polyclonal sera. Such polyclonal sera are composed of many individual monoclonal antibodies. Consequently, these data suggest that there are monoclonal antibodies that bind and neutralize multiple EVs. The identification of these cross-reactive, cross-neutralizing antibodies are the focus of current research.

## MATERIALS AND METHODS

### Ethics statement.

All experiments were performed in accordance with guidelines from the Guide for the Care and Use of Laboratory Animals of the NIH. Protocols were reviewed and approved by the Institutional Animal Care and Use Committee (IACUC) at Columbia University School of Medicine (assurance number AC-AABM2561).

### Antibodies.

Recombinant human anti-poliovirus antibody A12 was kindly provided by Konstantin Chumakov (Center for Biologics Evaluation, Food and Drug Administration). Rabbit anti-poliovirus polyclonal sera was generously shared by Andrew Macadam (National Institute for Biological Standards, United Kingdom).

### Cells and mice.

Rhabdomyosarcoma (RD), Vero, murine fibroblasts producing the human poliovirus receptor (59), and rhesus monkey kidney epithelial cells (LLC-MK2) were grown in Dulbecco’s modified Eagle’s medium (Invitrogen, Carlsbad, CA), 10% fetal calf serum (Atlas Biologicals, Fort Collins, CO), and 1% penicillin-streptomycin (Invitrogen). HeLa cells were grown in Dulbecco’s modified Eagle medium (Invitrogen, Carlsbad, CA), 10% calf serum (HyClone, Logan, UT), and 1% penicillin-streptomycin (Invitrogen).

C57/Black6 mice were bred in a specific-pathogen-free facility at Columbia University Medical Center.

### ELISA binding assays.

ELISAs were done in flat-bottomed microtiter plates (Nunc Maxisorb, Fisher, 44-2404-21). The wells were coated either with 10^5^ PFU of virus diluted in 100 mM bicarbonate/carbonate buffer (pH 9.6) or with 100 ng of anti-EV-D68 antibody diluted in 1× phosphate-buffered saline and incubated at 4°C overnight. Unbound virus or capture antibody was removed, and wells were washed using 1× Tris buffered saline (TBS)-Tween 20 at room temperature and incubated in blocking solution of 6% fetal bovine serum in 1× TBS-Tween 20 at room temperature for 3 h. If antibody coated, 10^5^ PFU of virus was diluted in 1× TBS-Tween 20, incubated at 4°C overnight, and unbound virus was removed as described above. Serial 2-fold dilutions of polyclonal sera or purified recombinant human monoclonal antibody in 1× TBS-Tween 20 were added, and the wells were incubated at 4°C overnight. Unbound antibody was removed, and the wells were washed using 1× TBS-Tween 20 and incubated in the presence of the appropriate F_c_ specific IgG-conjugated horseradish peroxidase-conjugated secondary (Invitrogen), diluted 1 to 10,000, in blocking solution for 90 min. The wells were washed, and antibody binding was detected by the addition of the OptEIA substrate (BD Biosciences). The reaction was quenched with the addition of 2 M sulfuric acid, and emission was assessed at 520 nm following excitation at 470 mn using a Synergy 2 (BioTek).

### Murine polyclonal sera.

Prior to immunization, 10^6^ PFU of the appropriate virus was mixed 1:1 (v:v) with Freund’s complete adjuvant (Millipore Sigma). Three-week-old male and female C57/Black6 mice were intraperitoneally immunized with adjuvanted virus at weeks 0, 3, 6, 9, and 12. Blood was collected by submandibular bleeding at weeks 2, 5, 8, 11, and 14 after immunization in EDTA-coated Eppendorf tubes, and cells were removed by centrifugation. Polyclonal sera were pooled prior to analysis.

### Plaque assay.

Cells (RD for EV-D68, EV-D94, and EV-D111; HeLa for poliovirus, HRV 1A, 2, 14, 16, 39, CVA24v, and echovirus 1; LLC-MK2 for echoviruses 2, 9, and 20, and EV-70 [RMK]) were seeded on 60-mm plates for approximately 70% confluence at the time of plaquing. Next, 100-μl portions of serial 10-fold virus dilutions were incubated with cells for 1 h at 37°C. Two overlays were added to the infected cells. The first overlay consisted of 2 mL of Dulbecco’s modified Eagle’s medium (DMEM), 0.8% Noble agar, 0.1% bovine serum albumin, 40 mM MgCl_2_, and 10% bovine calf serum. After solidification, a second liquid overlay was added that was composed of DMEM, 0.1% bovine serum albumin, 40 mM MgCl_2_, 0.2% glucose, 2 mM pyruvate, 4 mM glutamine, and 4 mM oxaloacetic acid. The cells were incubated at 37°C for 4 to 6 days and developed by using 10% trichloroacetic acid and crystal violet.

### Viruses.

Enterovirus D68 isolates (EV-D68): 18947 (947, 2014), 18949 (949, 2014), 18952 (952, 2014), 18953 (953, 2014), 18956 (956, 2014), 23209 (209, 2018), and 23263 (263, 2018) were obtained from BEI Resources. Rhyne EV-D68 (1962) isolate was kindly provided by Shigeo Yagi (California Department of Public Health, Richmond, CA). The NY68 (2009) isolate of EV-D68 was from the Lipkin collection. The Fermon isolate of EV-D68 (1962), echoviruses 1, 2, 9, and 30, and human rhinoviruses 1A, 2, 14, 16, and 39 were purchased from ATCC (American Tissue Culture Collection, Manassas, VA). Enteroviruses D94 and D111, and the Coxsackievirus A24 variant were generously given by Terry Fein Fan Ng and M. Steve Oberste (Division of Viral Diseases, Picornavirus, Centers for Disease Control and Prevention). The DNE variant of EV-D70, which was adapted for growth in HeLa cells, was used (60). All viruses were propagated and assayed in RD, HeLa, or LLMCKK2 cells. Viral titers were determined by plaque assay.

### Virus neutralization assay.

Murine polyclonal sera or recombinant human monoclonal antibodies were diluted serially 2-fold in Dulbecco’s phosphate-buffered saline (PBS) and added to 10^4^ PFU of virus. Virus-antibody mixture was rotated end-over-end for 1 h at room temperature. Neutralization of infectivity was assayed by plaque assay.

### Data analysis.

GraphPad Prism software was used to analyze all data. Log_10_-transformed titers were used for graphing the results of plaque assays.
